# Tuberculosis Presenting as Chronic Monoarthritis: A Case Study

**DOI:** 10.7759/cureus.39430

**Published:** 2023-05-24

**Authors:** Irene Pinto, Vilma Marques, Lúcia Dias

**Affiliations:** 1 Department of Physical Medicine and Rehabilitation, Centro Hospitalar Trás-os-Montes e Alto Douro, Vila Real, PRT; 2 Department of Physical Medicine and Rehabilitation, Centro Hospitalar de Trás-os-Montes e Alto Douro, Vila Real, PRT

**Keywords:** physical medicine and rehabilitation, total knee arthroplasty, knee osteoarthritis, pigmented villonodular synovitis, mycobacterium tuberculosis

## Abstract

*Mycobacterium tuberculosis* infection remains a common disease in developing countries with the potential to involve the osteoarticular system. The authors report a case of knee arthritis due to tuberculosis (TB) in a 34-year-old woman. The patient presented with pain and swelling of the right knee as major complaints, without a history of respiratory symptoms. Magnetic resonance imaging (MRI) demonstrated a marked joint effusion, involving synovial tissue with cartilaginous lesion compatible with pigmented villonodular synovitis (PVNS). After several physiotherapy courses without significant relief, total knee arthroplasty was proposed. Two months after surgery and rehabilitation, symptoms did not completely resolve, with limited active range of motion. Microbial bone biopsy culture at the time of the arthroplasty revealed a TB infection.

Due to the rarity and clinical nonspecificity of TB bone manifestations, early diagnosis may be challenging. Yet, attempted diagnosis and prompt pharmacological intervention are paramount to improve outcomes.

## Introduction

Tuberculosis (TB) is still a significant cause of morbidity and mortality worldwide, with the lung being the primarily affected organ; however, it can also affect extrapulmonary sites.

The skeletal system can be involved, predominantly at the spine and at large joints (sacroiliacs, hips, and knees) [1,2]. The incidence of knee joint TB is low and primarily occurs in underdeveloped countries. Besides, patients with a weakened immune system are more susceptible to latent TB of the knee joint. The most catastrophic complication of bone TB is the progression of bone destruction, potentially leading to a catastrophic outcome such as major amputation.

In general, osteoarticular TB involvement remains hidden for long periods, leading to a delay in the differential diagnosis [3]. As clinical manifestations of joint TB are atypical and vary widely, accurate diagnoses during the early stages of the disease remain difficult. In particular, knee joint TB may be easily misdiagnosed with primary knee osteoarthritis or other diseases, such as pigmented villonodular synovitis (PVNS) [4]. Both previous conditions present with functional restriction of the knee and clinically as monoarthritis with painful swelling for several months to years [5]. Differential diagnosis may be investigated by conventional X-rays, computed tomography (CT), or MRI to assess the articular surface damage, which could present as synovitis, osteopenia, and marginal erosions [6]. However, the sensitivity and specificity of these tests are highly variable. Furthermore, the standard treatment of TB arthritis and PVNS is different [5]. Therefore, a correct diagnosis is of utmost importance for the outcome.

## Case presentation

**Figure 1:**
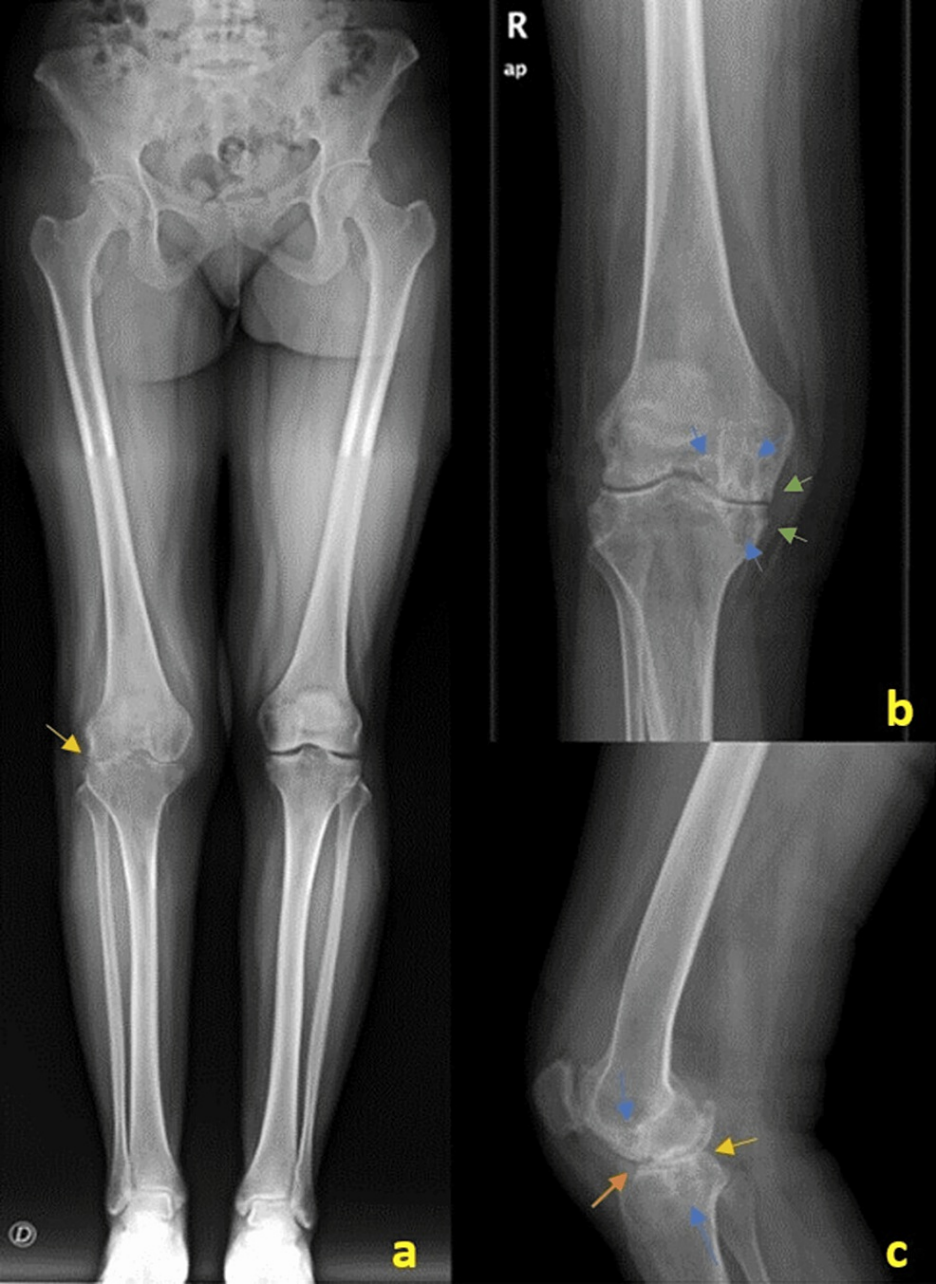
Lower limb X-rays. (a) Lower limb, (b) anteroposterior, and (c) lateral views of the right knee X-rays with signs of osteoarthritis, showing asymmetric narrowing of the articular joint space (yellow arrows) associated with articular surface irregularity (orange arrow), subchondral bone cysts (blue arrows), and discrete marginal osteophytes (green arrows). There is also (a) discrete valgus deformity.

A 34-year-old woman, presented to the outpatient Orthopedic clinic in 2018, with major complaints of pain, swelling, and restricted mobility in her right knee for seven years. The pain was characteristically exacerbated after strenuous activities, without any systemic manifestations (including respiratory) or trauma history. Physical examination reported the presence of swelling and tenderness through palpation of the right knee with impaired proper motions. X-rays showed an asymmetric narrowing of the involved joint space of the right knee (Figure 1). Systemic examination was unremarkable. As relevant background, the patient had a clinical diagnosis of PVNS at the age of 17 years, without documented surgical treatment.

Between 2018 and 2020, successive courses of physical therapy interventions were performed, with slight symptomatic improvement. Single short cycles of anti-inflammatory drugs were also used to manage the inflammatory process.

**Figure 2:**
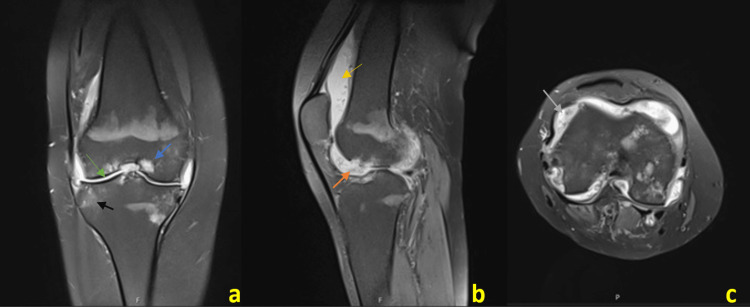
Right knee MRI. Fat-Saturated Proton Density MRI: (a) coronal, (b) sagittal, and (c) axial images of the right knee showing signs of osteoarthritis with a diffuse thickness reduction of the cartilage surfaces of the femorotibial compartments (green arrow), subchondral bone cysts (blue arrow), and osteochondral defect of the lateral femoral condyle (orange arrow), associated with areas of bone marrow edema (black arrow), diffuse joint effusion (yellow arrow), and mild synovial hypertrophy (gray arrow).

In September 2021, due to worsening pain and gait and range-of-motion (ROM) impairments, the patient presented to the emergency department. On examination, the right knee presented with increased swelling and crepitus, accompanied by several ROM restrictions. MRI revealed features of knee osteoarthritis with effusion and synovitis associated with cortical and trabecular erosions in the femorotibial compartment, leading to a probable association with PVNS (Figure 2).

A few months later, total right knee arthroplasty with patellofemoral replacement was performed (with intraoperative bone biopsy) and a new course of physical therapy was started immediately. Despite all the efforts, the failure in functional recovery was evident, as the right knee would not flex below 70°, nor extended above −20°, constantly with associated pain. The histopathologic examination of the tissue showed necrotizing granulomatous inflammation, and eight weeks after surgery, the culture of the bone biopsy revealed the presence of *Mycobacterium tuberculosis*. A regiment of anti-TB chemotherapy was instituted for 12 months. Pharmacotherapy concluded, and the patient already had no clinical symptoms, except slight ROM limitations.

## Discussion

TB remains a significant health issue in developing countries and can manifest in extrapulmonary sites, including the osteoarticular system. We reported the case of a 34-year-old patient with knee TB. Painful knee with the presence of abscess (16%) and sinus (42%) [7] are common findings. In this case, our patient presented progressive swelling and stiffness in the right knee and intermittent pain complaints without systemic manifestations.

Although radiological findings obtained in knee X-rays are not pathognomonic for TB infection, some radiographic features, including juxta-articular osteoporosis, peripheral osseous erosion, and gradual narrowing of the intra-articular space, are described, called triad of Phemister [8]. Especially at an early stage, these findings may mimic other osteoarticular lesions, as in the patient of this study, leading to the equivocal diagnosis of knee osteoarthritis.

In addition to cartilage destruction, TB infection leads to inflammatory and proliferative synovitis, joint effusion, and bone marrow edema on MRI, similar to PVNS [9]. Knee TB and PVNS may both present as monoarticular arthritis, with painful swelling for several months to years. In our case, the suspicion of PVNS and the high degree of joint destruction led to total arthroplasty of the right knee.

After hospital discharge, enhanced rehabilitation should aim at reducing pain and swelling, regaining muscle strength, and recovering knee ROM [10]. In case of insufficient knee ROM recovery associated with pain on movement, even after several weeks after surgery, especially in young people, it should raise suspicion and lead to a different diagnosis. Two months after surgery, a definitive diagnosis of knee TB infection relied on positive culture findings of bone biopsy, excluding alternative hypotheses, such as PVNS [11].

The course of treatment after the detection of TB infection in total knee arthroplasty is ambiguous. According to the literature, anti-TB drug treatment alone (for 12-18 months) is usually preferred with a high success rate, whereas the surgical treatment is reserved for specific indications and mostly to treat complications [4,11]. The timing and severity of infection will be decisive in preserving the prosthesis, with revision and fusion surgery providing alternative options [12]. The decision of prosthesis retention relates to the fact that TB rarely results in significant biofilm formation, owing to its poor adherence to metal when compared to the usual suspects, such as* Staphylococcus aureus* [13].

Although a case report, the authors of this study believe that highlighting institutional experience and difficulties dealing with rare diagnostics represents a didactic instrument to the medical community.

## Conclusions

The osteoarticular manifestation of TB is a rare condition and its diagnosis requires a high level of pretest suspicion, especially in cases of absence of pulmonary symptoms and previous history of infection. Misdiagnosis is frequent, and PVNS may appear as one of the diagnostic hypotheses. TB infection can lead to irreversible joint damage requiring total knee replacement, with articular restriction and pain persisting after surgery. The delay in restoring functional status suggests an alternative diagnosis, and TB should be remembered for every infection of the knee joint.
